# Identification of potential proteins translated from circular RNA splice variants

**DOI:** 10.1016/j.ejcb.2023.151286

**Published:** 2023-01-10

**Authors:** Aniruddha Das, Tanvi Sinha, Smruti Sambhav Mishra, Debojyoti Das, Amaresh C. Panda

**Affiliations:** a Institute of Life Sciences, Nalco Square, Bhubaneswar 751023, Odisha, India; b School of Biotechnology, KIIT University, Bhubaneswar 751024, India

**Keywords:** Circular RNA, Splice variants, Alternative splicing, Backsplicing, Cap-independent translation

## Abstract

Circular RNAs (circRNAs) are covalently closed RNA molecules generated from precursor RNAs by the head-to-tail backsplicing of exons. Hundreds of studies demonstrated that circRNAs are ubiquitously expressed and regulate cellular events by modulating microRNA (miRNA) and RNA-binding protein (RBP) activities. A few circRNAs are also known to translate into functional polypeptides regulating cellular physiology. All these functions primarily depend on the full-length sequence of the circRNAs. CircRNA backsplice junction sequence is the key to identifying circRNAs and their full-length mature sequence. However, some multi-exonic circRNAs exist in different isoforms sharing identical backsplice junction sequences and are termed circRNA splice variants. Here, we analyzed the previously published HeLa cell RNA-seq datasets to identify circRNA splice variants using the *de novo* module of the CIRCexplorer2 circRNA annotation pipeline. A subset of circRNAs with splice variants was validated by the circRNA-rolling circle amplification (circRNA-RCA) method. Interestingly, several validated circRNAs were predicted to translate into proteins by the riboCIRC database. Furthermore, polyribosome fractionation followed by quantitative PCR confirmed the association of a subset of circRNAs with polyribosome supporting their protein-coding potential. Finally, bioinformatics analysis of proteins derived from splice variants of *circCORO1C* and *circASPH* suggested altered protein sequences and structures that could affect their physiological functions. Together, our study identified novel circRNA splice variants and their potential translation into protein isoforms which may regulate various physiological processes.

## 1 Introduction

CircRNAs are one class of closed-loop single-stranded RNA molecules generated by the head-to-tail backward splicing of precursor RNA molecules. The first report in the 1970s showed that a circular form of RNA was found in viroids (Sanger et al., 1976). Afterward, circRNAs were also observed in the cytoplasm of eukaryotic cells by electron microscopy (Hsu and Coca-Prados, 1979). Initially, they were thought to be the result of splicing errors but were later shown to have a possible function in testis-specific circRNA originating from the *Sry* gene (Capel et al., 1993). The development of circRNA enrichment strategies followed by high-throughput RNA sequencing and analysis with advanced circRNA analysis tools discovered thousands of circRNAs and helped in their quantification in different cells or biological conditions (Xia et al., 2017; Salzman et al., 2012; Zhang et al., 2016). The tissue-specific expression of these circRNA transcripts was also observed in many cases (Xia et al., 2017). Unlike linear RNAs, circRNAs lack poly-A tailing and 5′ cap and are predominantly found in the cytoplasm (Hsu and Coca-Prados, 1979; Salzman et al., 2012; Jeck et al., 2013; Memczak et al., 2013). Most circRNAs are back-spliced from protein-coding genes and usually contain single or multiple exonic sequences that are also present in their linear counterpart transcripts (Guo et al., 2014). However, some circRNAs may contain altered exons or introns in the mature circRNA due to alternative splicing during circRNA biogenesis (Zhang et al., 2016; Zheng et al., 2019). Although most circRNAs are expressed in low quantities, circRNAs show altered expression in response to physiological changes or disease conditions. Until now, circRNAs have been shown to have regulatory roles in developing diseases like cancer, cardiovascular diseases, diabetes mellitus, and neurodegenerative diseases (Verduci et al., 2021; Chen et al., 2022; Maass et al., 2017). CircRNAs, once thought to be junk, are now reported to regulate various cellular pathways by acting as a sponge for miRNAs and proteins, thereby hindering the gene regulatory functions of these miRNAs and proteins (Memczak et al., 2013; Panda, 2018; Das et al., 2021; Dudekula et al., 2016).

Although a majority of circRNAs are grouped under the class of noncoding RNA molecules, recent studies highlighted the translation potential of circRNAs (Jeck et al., 2013; Memczak et al., 2013; Schneider et al., 2016; Sinha et al., 2022; Yang et al., 2017; Fan et al., 2022; Chen et al., 2021). Indeed, a few studies discovered the functional proteins translated from circRNAs through cap-independent mechanisms (Sinha et al., 2022). However, despite extensive explorations, circRNA splice variants’ identity and translation products are not well explored. This study used the *de novo* circRNA annotation tool to identify transcriptome-wide circRNA splice variants in HeLa cell RNA-seq data (Gao et al., 2016). In addition, we validated the expression of a subset of circRNA splice variants using our previously published circRNA-RCA method (Das et al., 2019). Furthermore, we identified potential peptides that could be translated from circRNA splice variants. Together, our findings vastly expand our current knowledge of circRNAs and the complexity of proteins coded by human transcriptome.

## 2 Methods

### 2.1 circRNA analysis

Previously published Poly(A)+ RNA-seq and RNase R treated RNA-seq data from HeLa cells (SRA ID: SRP049453) were downloaded and converted from SRA to fastq using SRAtookit v2.9.6 (Supplementary Fig. S1A) (Gao et al., 2016). The quality of raw RNA-seq data was analyzed using FastQC (v0.11.8) software. CircRNAs showing expression in HeLa cell samples and their alternative splicing variants were identified using the annotation, and *de novo* modules of CIRCexplorer2 v2.3.6 taking alignment output from Tophat-fusion for PolyA depleted RNA-seq and Tophat (v2.1.0) for Poly(A)+ RNA-seq (Supplementary Table S1) (Zhang et al., 2016). The mature spliced sequence of the identified circRNAs in HeLa cells was retrieved from hg38 genome assembly using BEDtools (Quinlan and Hall, 2010). A subset of abundant circRNAs with more than 20 read numbers was selected to validate circRNA splice variants and further analysis.

### 2.2 Cell culture, RT-PCR, Sanger sequencing, RNase R treatment, and quantitative (q)PCR

Total RNA was isolated from growing HeLa cells using TRIzol (Thermo Fisher Scientific) followed by reverse transcription using the High-Capacity cDNA Reverse Transcription kit (Thermo Fisher Scientific) following the manufacturer’s instructions. PCR amplification of target circRNAs was performed using a PCR program with denaturation for 2 min at 95 ° C, followed by 40 cycles of 5 s denaturation at 95 ° C and annealing/extension for 20 s at 60 ° C using DreamTaq PCR master mix and circRNA-specific divergent primers (Supplementary Table S2). After amplification, the PCR products were resolved on a SYBR Gold stained 2.5 % agarose gel showing the amplification of specific products, followed by purification of PCR products and Sanger sequencing to confirm the presence of the unique backsplice junction (BSJ) sequence of circRNAs. Quantitative (q)PCR analysis of target RNAs was performed with the same PCR program as described above on a QuantStudio realtime PCR system (Thermo Fisher Scientific) using specific primer pairs and [2x] PowerUp SYBR Green Master Mix (Thermo Fisher Scientific). The target RNA levels in RT-qPCR were calculated using the delta-CT method normalized to *GAPDH* mRNA.

### 2.3 Identification of circRNA splice variants by circRNA-RCA

Total RNA from HeLa cells was treated with RNase R to enrich the circRNA population using the previous protocol (Das et al., 2019). Then, the enriched circRNA pool was reverse transcribed with Maxima H-minus reverse transcriptase (Thermo Fisher Scientific) to make the full-length cDNA through the rolling circle amplification mechanism. The tandem repeats of full-length cDNA were used for PCR amplification with the PCR setup of 95 ° C for 2 min, followed by 40 cycles of 5 s at 95 ° C, 20 s at 58 ° C, and 60 s at 72 ° C using full-length forward and reverse primers and DreamTaq master mix as described in our previous publication (Das et al., 2019). The full-length circRNA PCR products were resolved on a 2.5 % agarose gel stained with SYBR Gold dye, followed by gel purification of specific circRNA bands and Sanger sequencing using one of the full-length primers used for amplification, revealing the full-length sequences of circRNA splice variants with same backsplice junction sequence.

### 2.4 Computational tools used for prediction of RNA structures, ORFs, IRES, m6A sites, protein structures, and domain analysis

The circRNA sequences used in the study were taken from the CIRCexplorer2 analysis of HeLa cell RNA-seq data as described above. To find the known circRNAs in our HeLa cell circRNA, the human circRNA list was downloaded from circAtlas 2.0 and compared with our HeLa cell circRNA analysis (http://159.226.67.237:8080/new/index.php) (Wu et al., 2020). The RNA secondary structures were predicted by mFold web server using default parameters (http://www.unafold.org/mfold/applications/rna-folding-form.php) (Zuker, 2003). The presence of an internal ribosomal entry site (IRES) on the *circASPH* sequence was checked on the circRNADb database (http://reprod.njmu.edu.cn/cgi--bin/circrnadb/circRNADb.php) (Chen et al., 2016). The HuR binding sites on specific circRNA splice variants were predicted by the CircInteractome web tool (https://circinteractome.nia.nih.gov/rna_binding_protein.html) (Dudekula et al., 2016). In addition, RBP sites on circRNAs were also predicted using the RBPmap web server (http://rbpmap.technion.ac.il/) (Paz et al., 2014). N6-methyladenosine (m6A) sites on circRNA splice variant sequences were predicted by the SRAMP prediction server using the mature RNA sequence mode and considering the RNA secondary structure (http://www.cuilab.cn/sramp) (Zhou et al., 2016). The full-length sequence of each circRNA splice variant was inserted thrice in succession to find the open reading frames (ORFs) crossing the backsplice junction using the NCBI ORF Finder web tool (https://www.ncbi.nlm.nih.gov/orffinder/) and GeneRunner software. The list of human circRNAs potentially translating into proteins was downloaded from the riboCIRC database (http://www.ribocirc.com) (Li et al., 2021a). The potential peptide sequences crossing the circRNA backsplice junction were predicted using the GeneRunner tool and matched with riboCIRC data. The peptide sequences crossing the back-splice junction in the circRNA splice variants were taken for structural analysis. The structure of predicted polypeptides encoded by circRNA splice variants was determined using Alpha Fold 2 with default parameters (https://colab.research.google.com/github/sokrypton/ColabFold/blob/main/beta/AlphaFold2_advanced.ipynb) (Jumper et al., 2021). InterProScan was used to identify functional protein domains reported by various prediction tools such as Pfam, Phobius, and PANTHER in the circRNA-derived polypeptides (https://www.ebi.ac.uk/interpro/search/sequence/) (Quevillon et al., 2005).

### 2.5 Polysome fractionation analysis

Polysome profiling of HeLa cells was performed as described previously (Panda et al., 2017). Briefly, the HeLa cells were treated with or without 0.6 mM puromycin for 3 h followed by 10 mins treatment with cycloheximide (200 μg/mL). The treated cells were washed with [1x] PBS and lysed in polysome extraction buffer (PEB). The cell lysate was centrifuged for 10 min at 10,000 RPM at 4 °C, followed by the supernatant collection and loading on a 10–50 % sucrose gradient. Next, the sucrose gradient was centrifuged at 1,90,000 x g (SW41Ti rotor) for 2.5 h at 4 °C in an ultracentrifuge machine (Optima XPN-100, Beckman Coulter). After ultracentrifugation, the sucrose gradient was fractionated into 12 separate fractions (from top to bottom) using the polysome fractionator (BioComp Gradient Station). The fractions 1–2, 3–4, 5–6, 7–8, 9–10, and 11–12 were pooled to have 6 fractions for RNA isolation and RT-qPCR analysis. The RNA and cDNA from each fraction were prepared as described above. The distribution of housekeeping *GAPDH* mRNA and target circRNAs in each fraction were studied by RT-qPCR analysis.

### 2.6 HuR RNA immunoprecipitation, m6A Pulldown and dot blot assay

The association of circular RNAs with HuR in HeLa cells was analyzed by RNA immunoprecipitation (RIP) of HuR, as described previously (Abdelmohsen et al., 2017). Briefly, HeLa cell lysates were prepared in PEB supplemented with RNase inhibitor and [1x] protease inhibitor. The lysate was incubated with protein G Dynabeads (Invitrogen, 10004D) coated with control IgG (CST, 2729 s) or HuR antibody (Abcam, ab200342) for 2 h at 4 °C followed by washing and isolation of RNA associated with the antibody-conjugated beads. The RNA was subjected to RT-qPCR analysis of circRNAs. Analysis of potential N6-Methyladenosine (m6A) sites on *circCORO1C* and *circASPH* using SRAMP prediction server revealed possible m6A sites on these circRNAs. So, to validate the m6A modifications of circRNAs experimentally, we performed m6A pulldown assay followed by RT-qPCR analysis. Briefly, 5 μg of HeLa total RNA was incubated for 2 h at 4 ° C with Protein-G Dynabeads conjugated to either m6A antibody (Abcam, ab208577) or Anti-Rabbit IgG antibody (CST, 2729 s). The beads were washed twice in ice-cold NT2 buffer, followed by RNA isolation, dot blot, and RT-qPCR analysis. Total RNA was used for dot blot analysis following a previously published protocol (Shen et al., 2017). Briefly, 50 ng of total RNA was subjected to denaturation, followed by 2 μl drop on a Hybond N + membrane and crosslinking. After crosslinking, the m6A primary antibody was applied to the RNA-bound membrane, followed by washing and incubation with appropriate secondary antibody. The m6A RNA signals were developed with chemiluminescence. The relative enrichment of the target circRNAs in m6A RNA was calculated by RT-qPCR analysis.

## 3 Results

### 3.1 Bioinformatic identification of circRNA splice variants in HeLa cells

**Fig. 1:**
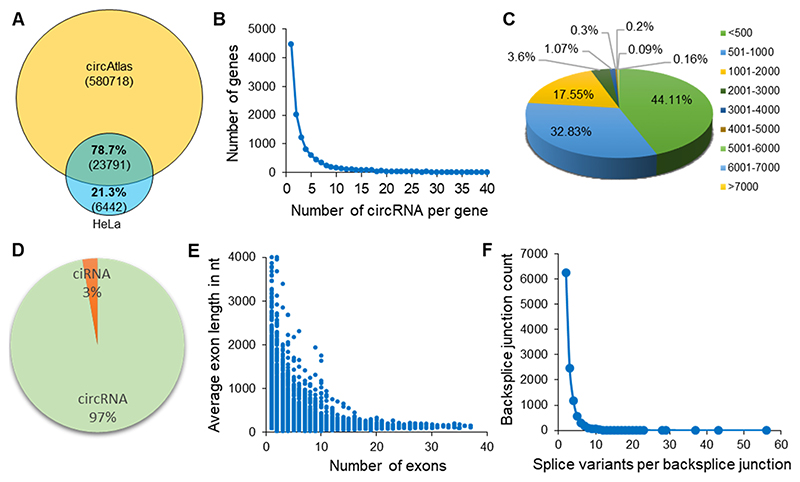
Characteristics of circRNAs and their splice variants in HeLa cells. A. Venn diagram represents the known and novel backsplice junctions in HeLa cells using CIRCexplorer2 pipeline compared to human circRNAs reported by circAtlas. B. Number of genes plotted against the number of circRNAs generated from a single gene in HeLa cells. C. Pie chart showing the percentage of circRNAs of various lengths expressed in HeLa cells identified using CIRCexplorer2. D. Percentage of circular RNAs generated from exons (circRNAs) and introns (ciRNAs). E. Size distribution of exon length in circRNAs compared to the number of exons included during backsplicing. F. The number of circRNA splice variants generated from a particular backsplice junction.

We used circRNA-enriched HeLa cell RNA-seq data from a previous publication to identify circRNAs using the CIRCexplorer2 annotation pipeline (Supplementary Fig. S1A) (Gao et al., 2016). We identified a total of 52,527 circRNAs with 30,233 unique backsplice junctions. Of the 30,233 unique backsplice junctions, 23,791 were previously identified in circAtlas 2.0 (Fig. 1A) (Wu et al., 2020). A total of 11,682 genes were found to generate circRNAs, out of which most of the circRNA-producing genes produced 1–10 circRNAs (Fig. 1B). The HeLa cell circRNAs identified here were mostly less than 2000 nucleotides (nt) in length (Fig. 1C). Analysis of the sequence composition of circRNAs identified here found that 51,066 (97 %) are exonic circRNAs while the rest 1461 are ciRNAs originating from intronic regions (Fig. 1D). As shown in Fig. 1E, the average length of exons is longer in circRNAs with few exons and *vice versa*. We identified 11,207 unique backsplice junctions that generate two or more splice variants with different exon/intron combinations due to alternative splicing (Fig. 1F).

### 3.2 Validation of circRNAs and their splice variants

**Fig. 2:**
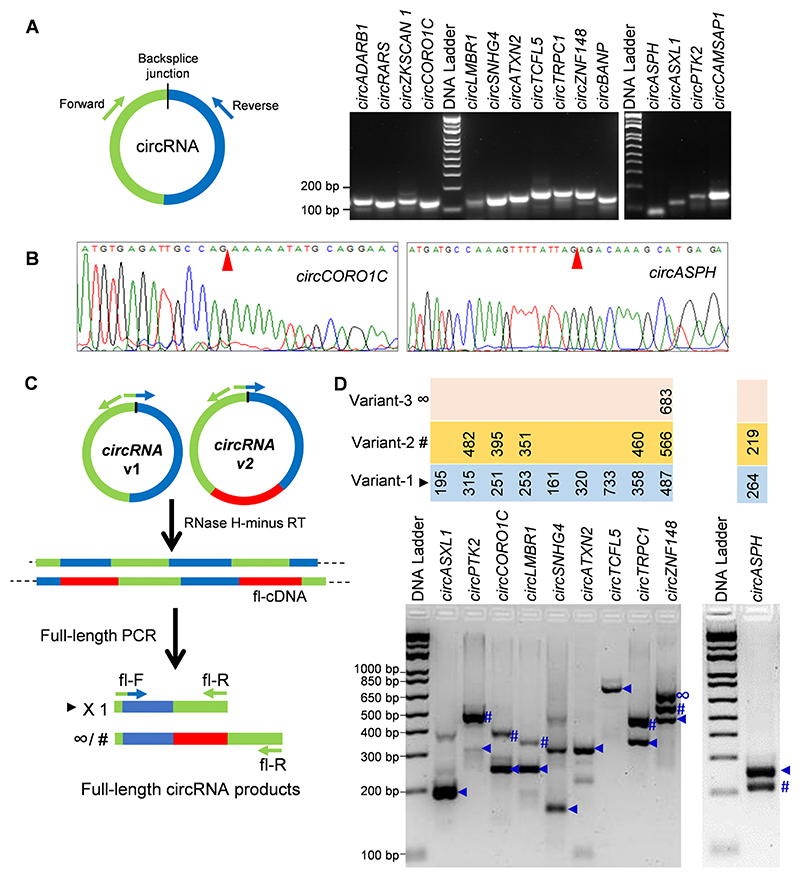
Validation of circRNAs and their splice variants in HeLa cells. A. Diagrammatic representation of divergent primer design (*left)* used to amplify the circRNA backsplice junction using RT-PCR followed by resolving the PCR products on a SYBR Gold stained 2.5 % agarose gel (*right*). B. Chromatogram shows circRNA backsplice junction sequence upon Sanger sequencing of the divergent primer-assisted PCR products. The red arrow points at the backsplice junction site C. Schematic representation of circRNA-RCA includes the full-length PCR primer design strategy, rolling circle amplification of cDNA, and PCR amplification of circRNA splice variants. D. The full-length circRNA PCR products were resolved on a 2.5 % agarose gel stained with SYBR Gold dye. The length of the variants verified by Sanger sequencing is mentioned at the top.

A subset of abundant circRNAs with a maximum length of 1500 nt, a maximum of 5 exons, and predicted splice variants were selected for RTPCR validation in HeLa cells using divergent primers (Table 1, Fig. 2A). The circRNAs, including *circCAMSAP1, circASXL1, circASPH, circADARB1, circRARS, circZKSCAN1, circPTK2, circCORO1C, circBANP, circLMBR1, circSNHG4, circATXN2, circTCFL5, circTRPC1,* and *circZNF148* were PCR amplified, and the amplicons were resolved on an agarose gel followed by gel elution and Sanger sequencing to verify the backsplice junction (Fig. 2A, B). However, we could confirm the back-splice junction amplification for all circRNAs tested here except *circPTK2* (Fig. 2B, Supplementary Fig. S1B). In addition, the primers targeting *circPTK2* amplified another *circPTK2* backsplice junction from the same locus of *PTK2* mRNA, probably due to its higher abundance.

Out of the above validated HeLa cell circRNAs, rolling circle amplification (RCA) of cDNA followed by full-length PCR and Sanger sequencing of the gel extracted PCR products revealed the presence of multiple splice variants only for 6 circRNAs, including *circASPH, circCORO1C, circTRPC1, circZNF148, circPTK2,* and *circLMBR1* [Fig. 2C, D]. However, the RCA method followed by Sanger sequencing revealed the presence of only one variant for *circASXL1, circATXN2, circSNHG4,* and *circTCFL5*. In addition, there were a few other full-length PCR product bands in the gel that could not be verified by Sanger sequencing. These PCR products could be either non-specific amplifications or concatamers of the variants formed due to repeats of full-length cDNAs generated during rolling circle amplification.

### 3.3 Identification of protein-coding HeLa circRNAs

**Fig. 3:**
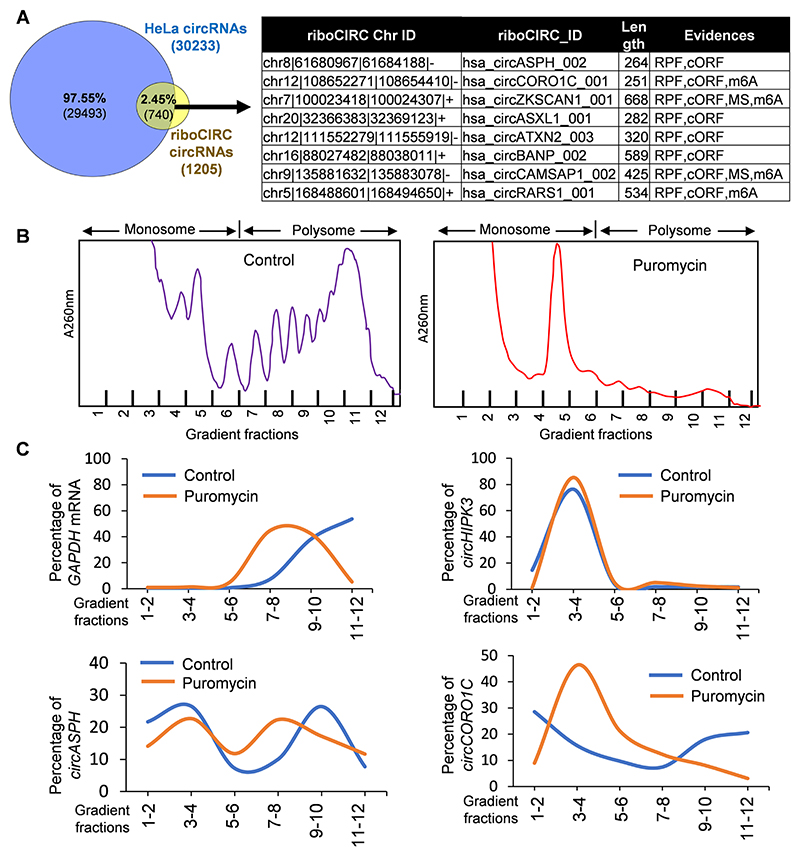
Translational potential of circRNAs. A. Venn diagram showing the HeLa circRNAs and translatable circRNAs reported in riboCIRC database. The right side table shows the validated HeLa circRNAs reported by riboCIRC database. B. RNA absorbance profile for the polyribosome fractionation of untreated (left) and puromycin treated (right) Hela cell extracts fractionated on a 10–50 % sucrose gradient. C. RT-qPCR analysis of different RNAs distributed upon polysome profiling showing the abundance of circRNAs in different fractions of the sucrose gradient with or without puromycin treatment. Data in *B* and *C* are representative of the 3 independent experiments.

To identify the potentially translatable HeLa cell circRNAs, we downloaded the human circRNAs with translation ability from the riboCIRC database (Supplementary Table S3). These circRNAs were predicted to code for proteins based on multiple evidences, such as the presence of ORFs, IRES, m6A modifications, ribosome protected fragments (RPFs), and mass spectrometric (MS) evidence of peptides generated from the backsplice junction (Li et al., 2021a). Fig. 3A shows eight validated circRNAs in HeLa cells, including *circASPH, circCORO1C, circATXN2, circBANP, circASXL1, circRARS, circZKSCAN1,* and *circCAMSAP1* were reported to be translated into proteins by riboCIRC database. However, circRNA-RCA analysis revealed only the expression of splice variants of *circCORO1C* and *circASPH* in HeLa cells.

For validating the translational ability of the candidate circRNAs, polysome profiling was performed on HeLa cell lysates. As shown in Fig. 3B, the control HeLa cell lysates showed nice polyribosome peaks, while the puromycin treatment disrupted the translating heavy polysomes and accumulated the monosomes. The 12 fractions collected from the sucrose gradient were combined to have a total of 6 fractions, where the first three fractions represent the ribosome-free fractions and monosomes, while the last 3 fractions containing translating heavy polyribosomes and were termed polysomes. RT-qPCR analysis of the sucrose gradient fractions revealed that the housekeeping *GAPDH* mRNA is mostly present in the polysome fractions in control cells, while puromycin treatment shifted the *GAPDH* mRNA towards light polysomes or monosomes, validating the fractionation of actively translating RNAs on the sucrose gradient (Fig. 3C). Interestingly, a small but significant percentage of *circASPH* and *circCORO1C* were found to be associated with translating polysomes in HeLa cells. Similar to actively translating *GAPDH* mRNA, *circASPH* and *circCORO1C* shifted towards monosomes in puromycin-treated HeLa cells, indicating their association with actively translating polyribosomes (Fig. 3C). As a negative control, abundant *circHIPK3* was mostly detected in the monosome fractions in HeLa cells.

### 3.4 Possible cap-independent translation of circRNA splice variants

**Fig. 4:**
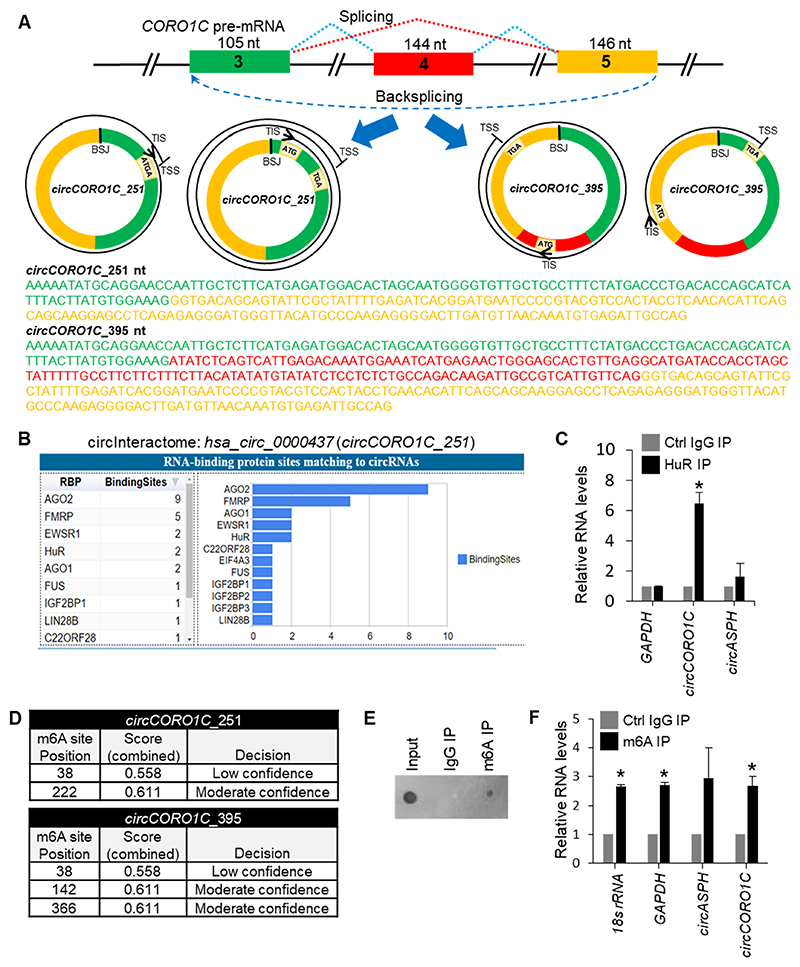
Characterization of potential circRNA peptides generated from *circCORO1C* splice variants. A. Diagrammatic representation showing biogenesis of *circCORO1C* splice variants, potential ORFs, and full-length sequences. B. HuR binding sites on *circCORO1C*_*251* (*hsa_circ_0000437*) predicted by circInteractome database. C. HuR IP in HeLa cell lysates followed by RNA isolation and RT-qPCR analysis of target circRNAs normalized to *GAPDH* mRNA. D. Prediction of m6A sites on *circCORO1C* splice variants with SRAMP web server. E. Dot blot analysis of RNAs in control IgG and m6A IP samples. F. RT-qPCR analysis of *circASPH* and *circCORO1C* in the m6A IP samples.

As shown in Fig. 4A, *CORO1C* pre-mRNA produces two circRNA splice variants with the backsplice junction coordinate chr12| 108652271|108654410|-. The *circCORO1C_251* and *circCORO1C_395* splice variants with different exon combinations were predicted to contain ORFs spanning the backsplice junction sequence. Similarly, *ASPH* pre-mRNA generated two *circASPH* splice variants of 219 and 264 nt length with the same backsplice junction at chr8|61680967| 61684188|-. The circRNA ORF prediction suggested the presence of an infinite ORF without any stop codon for both *circASPH_219* and *cicASPH_264* variants (Supplementary Fig. S2A). Analysis of the *circASPH* sequence in the circRNADb database revealed the presence of IRES sequence (Chen et al., 2016). The structure prediction using the mFold web server revealed secondary structures of *circASPH* IRES sequence regions, which could promote cap-independent translation (Supplementary Fig. S2B) (Zuker, 2003). RNA-binding proteins like HuR promote cap-independent translation mediated by IRES (Rivas-Aravena et al., 2009; Lin et al., 2015). Here, we could identify HuR binding sites on *circCORO1C and circASPH* using CircInteractome or RBPmap web servers (Fig. 4B, Supplementary Fig. S3A, S3B) (Dudekula et al., 2016; Paz et al., 2014). We performed HuR RIP assay followed by RT-qPCR analysis to experimentally validate the interaction of HuR with these circRNAs in HeLa cells. Although both circRNAs were predicted to bind HuR, we could detect the significant enrichment of *circCORO1C* in the HuR RIP samples in HeLa cells (Fig. 4C). In addition, m6A modification is also known to promote cap-independent translation of circRNAs (Yang et al., 2017). RNA sequences of *circASPH* and *circCORO1C* splice variants were predicted to contain a few m6A sites at 3–4 different positions by the SRAMP prediction server considering the RNA secondary structure (Fig. 4D, Supplementary Fig. S3C) (Zhou et al., 2016). Furthermore, *circCORO1C* was predicted to contain m6A modification by the riboCIRC database (Fig. 3A). RNA pulldown assay using m6A antibody followed by RT-qPCR analysis showed an enrichment of *circCORO1C* in m6A pulldown samples along with known m6A modified RNAs such as *18S* rRNA and *GAPDH* mRNA, confirming the presence of m6A modifications (Fig. 4E,F). Based on these bioinformatics and experimental evidence, including the presence of ORFs, RPFs, IRES, binding to HuR, m6A modifications, and association with translating polyribosomes suggests that *circCORO1C* and *circASPH* could be translated into proteins in the HeLa cells.

### 3.5 Potential function of the protein derived from circASPH and circCORO1C splice variants

**Fig. 5:**
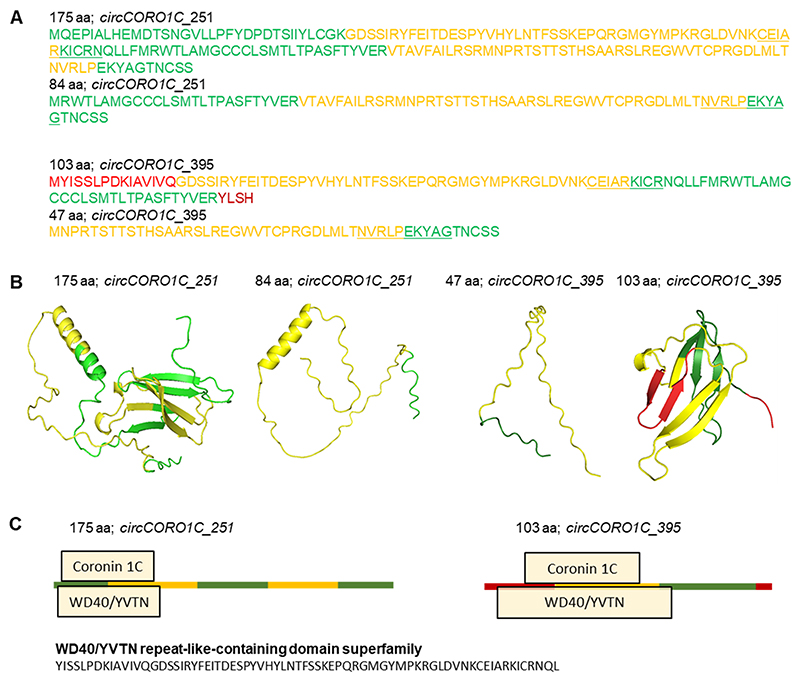
Characterization of potential peptides generated from *circCORO1C* splice variants. A. Backsplice junction spanning peptide sequence generated from different splice variants of *circCORO1C*. Different colors indicate different exons of the circRNA splice variants, and the underlined sequence marks the backsplice junction sequence. B. AlphaFold2 generated potential circRNA peptide structure coming from *circCORO1C* splice variants. C. InterProScan generated domain analysis of the *circCORO1C* splice variant peptides.

The presence of ORF and RPF data from the riboCIRC database and our analysis of m6A modifications and HuR binding sites suggested the potential translation of polypeptides from the *circCORO1C* splice variants. We identified the ORFs and peptide sequences generated from splice variants of *circCORO1C* (Fig. 5A). The *circCORO1C_251* splice variant is predicted to translate into two different polypeptides of 84 aa and 175 aa, while *circCORO1C_395* splice variants produce two polypeptides of size 103 aa and 47 aa. Furthermore, the 47 aa polypeptide generated from *circCORO1C* has been shown to function in angiogenesis in endometrial cancer cells (Li et al., 2021b). AlphaFold2 analysis of proteins derived from *circCORO1C* splice variants predicted the 3-dimensional structure of these circ-proteins (Fig. 5B). Domain prediction using InterProScan gives an integrated list of functionally relevant sites reported by various prediction tools such as Pfam, Phobius, and PANTHER. Interestingly, the *circCORO1C_251* translated 175 aa polypeptide, and *circCORO1C_395* translated 103 aa polypeptide contains Coronin-1C and WD40/YVTN repeat-like-containing domain superfamily whose function in directed cell migration and cell motility through its actin-binding property and as a protein-protein or protein-DNA interaction platform has already been reported (Fig. 5C) (Xu and Min, 2011; Chan et al., 2012; Williamson et al., 2014, 2015). However, the 84 aa and 47 aa polypeptides translated from *circCORO1C* contain no specific domain as predicted by InterProScan.

The ORF sequence of *circASPH_264* contains an extra 15 amino acids sequence compared to the *circASPH_219* variant (Supplementary Fig. S4A). We speculate that *circASPH* splice variants without a stop codon can translate into large-size proteins with repetitive sequences through the rolling circle translation. The 3-dimensional structure prediction of proteins derived from *circASPH_219* and *circASPH_264* using AlphaFold2 suggested a folded helical structure for both isoforms (Supplementary Fig. S4B). The predicted structure of proteins encoded by circular ASPH splice variants revealed the helical structure containing Aspartyl beta-hydroxylase/Triadin domain. Both *circASPH_219* and *circASPH_264* isoforms share overlapping regulatory domains with the linear ASPH protein (Supplementary Fig. S4C). Together these findings from the structural analysis and domain profile of the different peptides generated from *circCORO1C* and *circASPH,* suggest a possible function of these circ-proteins that might be supplementary to the function of the linear protein.

## 4 Discussions

Recently, genomic studies suggested that most of the human genome is transcribed into noncoding RNAs (Coffey et al., 2011; Hangauer et al., 2013). CircRNAs constitute a large family of covalently closed single-stranded RNA molecules generated from backsplicing (Memczak et al., 2013; Rybak-Wolf et al., 2015). Moreover, circRNAs are widely expressed in all eukaryotes, and some are conserved in the evolutionary tree (Rybak-Wolf et al., 2015). In addition, circRNAs often show altered expression during disease and development, indicating their physiological functions (Chen et al., 2022). In the last few years, circRNAs have emerged as novel regulatory RNA molecules. Like linear RNA alternative splicing, circRNAs also undergo alternative splicing to generate diverse circRNAs containing the same backsplice junction sequence but different combinations of spliced exons/introns (Zhang et al., 2016; Das et al., 2019). Although many reports suggested the extensive alternative splicing and generation of circRNA splice variants, most databases report only one variant for one circRNA based on the backsplice junction. Since mature full-length sequences of circRNA are critical for their functions, it is important to find the actual mature sequence of circRNAs before proceeding to downstream functional analysis.

In the present study, we identified hundreds of circRNA splice variants with the same backsplice junction sequence in HeLa cell RNA-seq data (Gao et al., 2016). Since the circRNA body sequence is shared with the linear host gene, the computational approaches to generate the full-length sequence of circRNAs could be error-prone. In our previous publication, we described the circRNA-RCA strategy to validate the internal parts of circRNA isoforms sharing the same backsplice junction (Das et al., 2019). As shown in Fig. 2, we could validate the expression of a few variants of a subset of abundant circRNAs using the circRNA-RCA approach (Das et al., 2019, 2022). The main advantage of circRNA-RCA is its ability to experimentally validate the expression of circRNA splice variants independent of computational predictions. Although circRNA-RCA analysis could not validate the expression of some splice variants identified for the tested circRNAs in RNA-seq analysis, that does not exclude that they may be expressed in low levels or under specific conditions of the cells.

Although most circRNA studies indicate their miRNA sponging activity in gene regulation, a few recent studies indicated the translation of circRNAs into functional polypeptides (Sinha et al., 2022). Furthermore, an increasing number of studies report the possible translation of proteins from circRNAs and their role in cellular physiology (Sinha et al., 2022). Since circRNAs do not possess 5′cap and poly-A sequences, it is translated through cap-independent mechanisms depending on the presence of IRES and m6A modifications (Yang et al., 2017; Fan et al., 2022). Interestingly, two recent reports suggested the pervasive translation of circRNAs through short sequences and structural elements acting as IRES for cap-independent translation (Fan et al., 2022; Chen et al., 2021). Furthermore, m6A modifications on circRNAs have been shown to drive cap-independent translation (Yang et al., 2017). However, the novel polypeptides translated from circRNA splice variants have not been explored extensively.

In this study, many circRNAs identified in HeLa cells were shown to have translation potential in the riboCIRC database (Li et al., 2021a). The riboCIRC database suggested the presence of ORF and RPF, indicating the potential translation of *circCORO1C* and *circASPH* by associating with translating ribosomes. Indeed, polyribosome fractionation assay with or without puromycin treatment suggested their association with translating ribosomes, indicating potential translation of *circCORO1C* and *circASPH* into peptides (Fig. 3). However, this does not rule out the possibility of circRNA co-sedimentation into the polysomes by directly or indirectly associating with translating mRNAs. In addition, the presence of IRES, m6A sites, and HuR binding sites on circRNA indicates the translation of circRNAs through cap-independent mechanisms. Although *circCORO1C* and *circASPH* are predicted to have HuR binding sites, other translation regulators, such as FMRP and AGO2, are also predicted to bind *circCORO1C*. The binding sites of AGO2 indicate the miRNA association with these circRNAs. The binding of HuR and FMRP with *circCORO1C* indicates a coordinated regulation of circRNA translation through the cap-independent mechanism. However, the actual interaction of these RBPs with *circCORO1C* and their role in translation needs to be validated experimentally.

Moreover, the ORF and RPF evidence from riboCIRC database and our data of polysome association, m6A sites, and HuR interaction indicated that *circCORO1C* and *circASPH* splice variants could be translated into polypeptides through cap-independent mechanisms (Fig. 3, Fig. 4, and Supplementary Fig. S3). Intriguingly, the polypeptides translated from *circCORO1C* splice variants contain Coronin-1C and WD40/YVTN repeat-like-containing domain superfamily, which may regulate cell migration and cell motility (Xu and Min, 2011; Chan et al., 2012; Williamson et al., 2014, 2015). In addition, the riboCIRC database also reported a 84 aa polypeptide generated from *circCORO1C_251*. Since circRNAs are covalently closed loop structures, they may contain multiple junction-spanning ORFs using different translation start codons in the same or different frames leading to the generation of multiple polypeptides from one RNA template (Orr et al., 2020). For example, the downstream ATGs in the *circCORO1C_251* can translate into another 84 aa polypeptide, which is the C-terminus sequence of the 175 aa polypeptide. A recent article reported the translation of a 47 aa polypeptide translated from *circCORO1C* that regulates angiogenesis in endometrial cancer cells (Li et al., 2021b). Although ORFfinder predicted the ORF coding for the 47 aa polypeptide only in *circCORO1C_395,* but the same polypeptide is also a part of the 175 aa polypeptide sequence translated from *circCORO1C_251.* Hence, the 47 aa polypeptide could be translated from both the *circCORO1C* variants by using alternative ORFs (Orr et al., 2020). The protein made from *circASPH* could have multiple repeats of the triadin domain, which is known to regulate calcium release and connect the reticulum to the microtubule network (Marty et al., 2009). This triadin domain is also found in the ryanodine receptor and calsequestrin binding proteins in the junctional sarcoplasmic reticulum of striated muscles (Goonasekera et al., 2007). However, we could not find mass spectrometry evidence supporting the peptide sequence generated from the backsplice junction of these circRNAs. In addition, we could not perform western blot analysis due to the unavailability of suitable antibodies for the specific detection of endogenous circ-proteins translated from *circCORO1C* and *circASPH*.

Although our data highlight the importance of circRNA splice variants and their translation products, there are a few limitations that could be improved. First, the circRNA splice variants identified from RNA-seq data using computational pipelines are far from complete. Novel low-abundant variants can be identified with better circRNA enrichment and deeper sequencing. Second, the peptide sequences predicted here are mostly based on indirect evidence of circRNA translation and computational predictions. Third, the structure and functions of the circ-proteins are hypothesized based on computational tools, which need experimental validation. Fourth, the *in vivo* expression of circ-proteins is not validated by mass spectrometry or western blot analysis. Finally, since antibodies specific to the circRNA-encoded proteins are unavailable, future studies using overexpression systems and custom antibodies against circ-proteins will reveal their expression and cellular mechanisms.

In conclusion, our study emphasizes that apart from validating the backsplice junction, the internal sequence of circRNAs must be validated to find the expression of possible splice variants of the same circRNAs, which might have different biological functions. Furthermore, we identified novel splice variants of circRNAs and their potential translation products. Together, these findings expand our knowledge and indicate the need for in-depth studies to explore the novel proteins translated from circRNAs which are mostly unknown and that will be helpful to deepen our understanding of circRNAs in human pathophysiology.

## Supplementary Material

Supplementary figures

Table S1

Table S2

Table S3

Table S4

## Figures and Tables

**Table 1:** List of validated circRNAs, length of splice variants, and their circAtlas IDs.

Unique backsplice junction ID	Gene Name	Splice variant length in nt (Exon count)	circAtlas IDs
chr21|45128401|45134832|+	ADARB1	290(2), 292(2), 293(2), 294(2), 296(2), 382(3), 506(4)	hsa-ADARB1_0010
chr8|61680967|61684188|-	ASPH	219(2), 264(3), 266(3), 333(4)	hsa-AC090094_0001
chr20|32366383|32369123|+	ASXL1	149(2), 192(2), 193(2), 195(3), 282(3)	hsa-ASXL1_0001
chr12|111552279|111555919|-	ATXN2	248(3), 320(4)	hsa-ATXN2_0002
chr16|88027482|88038011|+	BANP	407(4), 416(4)	hsa-BANP_0012
chr9|135881632|135883078|-	CAMSAP1	1024(2), 1033(2), 1446(1), 423(2), 425(2), 426(2), 587(3), 639(3)	hsa-CAMSAP1_0001
chr12|108652271|108654410|-	CORO1C	251(2), 274(3), 286(2), 395(3)	hsa-CORO1C_0003
chr7|156826604|156836885|-	LMBR1	253(3), 257(3), 267(3), 286(3), 331(3), 351(4), 371(4)	hsa-LMBR1_0004
chr8|140864311|140890769|-	PTK2	315(2), 482(3)	hsa-PTK2_0007
chr5|168488601|168494650|+	RARS	425(3), 478(4), 534(4)	hsa-RARS_0012
chr5|139278326|139279129|+	SNHG4	161(2), 249(2), 337(3), 493(2), 803(1)	hsa-SNHG4_0001
chr20|62854015|62860308|-	TCFL5	730(4), 733(4)	has-TCFL5_0001
chr3|142736378|142748460|+	TRPC1	358(2), 460(3)	hsa-TRPC1_0001
chr7|100023418|100024307|+	ZKSCAN1	578(3), 668(2)	hsa-ZKSCAN1_0001
chr3|125313307|125331238|-	ZNF148	487(3), 566(3), 604(4), 683(4)	hsa-ZNF148_0013

## Data Availability

Data will be made available on request.
