# Supplementary material for: Identification of potential proteins translated from circular RNA splice variants
Source: Eur J Cell Biol. Author manuscript; Available in PMC 2023 May 11. (PMC7614519; doi:10.1016/j.ejcb.2023.151286)
Supplement: Supplementary figures [file EMS175015-supplement-Supplementary_figures.pdf]

Identification of potential proteins translated from circular RNA splice variants

Aniruddha Das[1,2], Tanvi Sinha[1], Smruti Sambhav Mishra[1], Debojyoti Das[1], and Amaresh C. Panda[1,*]

[1] Institute of Life Sciences, Nalco Square, Bhubaneswar-751023, Odisha, India

[2] School of Biotechnology, KIIT University, Bhubaneswar-751024, India

***Correspondence**: Amaresh C Panda; Email: amaresh.panda@ils.res.in

Supplementary Tables: 4

Supplementary Figures: 4

Supplementary Tabe S1: HeLa cell circRNA annotation

Supplementary Table S2: Oligonucleotides used in the study

Supplementary Table S3: HeLa cell circRNAs in riboCIRC database

Supplementary Table S4: Domain analysis of circRNA-derived proteins using InterProScan

Supplementary Figure S1

A

Bio Project No. **PRJNA266072** (*Gao, et al. Nat Commun, 7, 12060*)				
**Sample name (HeLa cells)**	**Experiment ID**	**SRA ID**	**Bio Sample ID**	**SRA Run ID**
PolyA+ sequencing without RNase R treatment (polyA+/RNaseR-)	SRX1744883	SRP049453	SAMN03159253	SRR3479116
With RNase R treatment (RNaseR+)	SRX749241	SRP049453	SAMN03159253	SRR3476956
With RNase R treatment (RNaseR+)	SRX749241	SRP049453	SAMN03159253	SRR1636986
With RNase R treatment (RNaseR+)	SRX749241	SRP049453	SAMN03159253	SRR1636985

B

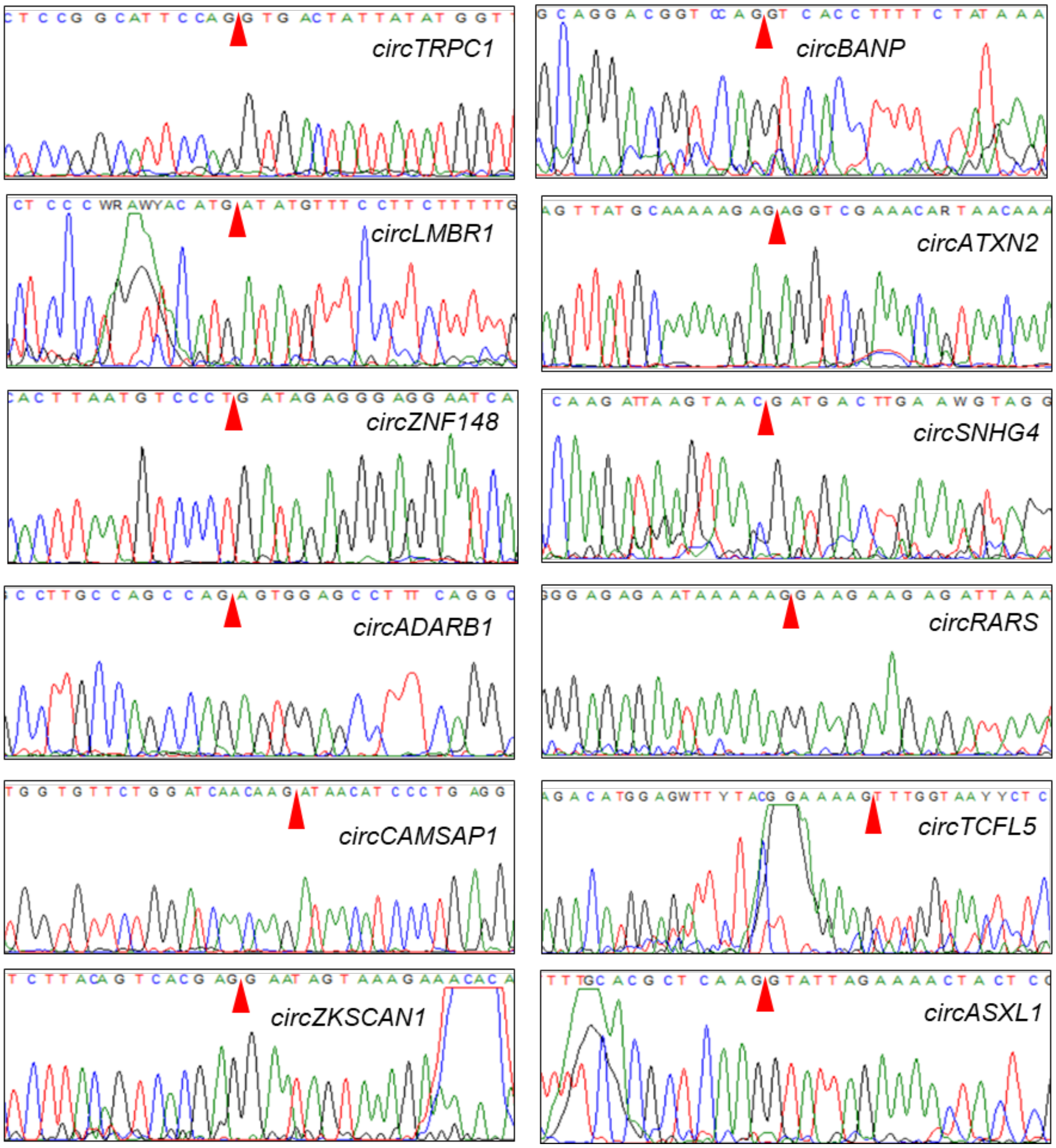

Supplementary Figure S1: Identification of circRNAs. **A.** RNA-seq datasets taken for identification of circRNAs in HeLa cells using CIRCexplorer2. **B.** Sanger sequencing results validate the presence of backsplice junction sequence of selected circRNAs.

Supplementary Figure S2

A

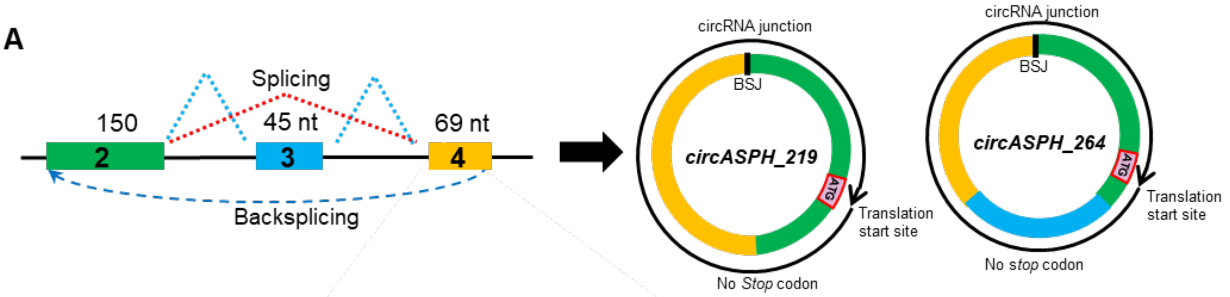

circASPH_219:AGACAAAGCATGGAGGACACAAGAATGGGAGGAAAGGCGGACTCTCAGGAACTTCATTCTTCACGT
GGTTTATGGTGATTGCATTGCTGGGCGTCTGGACATCTGTAGCTGTCGTTTGGTTTGATCTTGTTGACTATGAGGAAG
TTCTAGGAAAACTAGGAATCTATGATGCTGATGGTGATGGAGATTTTGATGTGGATGATGCCAAAGTTTTATTAG

circASPH_264:AGACAAAGCATGGAGGACACAAGAATGGGAGGAAAGGCGGACTCTCAGGAACTTCATTCTTCACGT
GGTTTATGGTGATTGCATTGCTGGGCGTCTGGACATCTGTAGCTGTCGTTTGGTTTGATCTTGTTGACTATGAGGAAG
TTCTAGCCAAAGCAAAGGACTTCCGTTATAACTTATCAGAGGTGCTTCAAGGAAAACTAGGAATCTATGATGCTGATG
GTGATGGAGATTTTGATGTGGATGATGCCAAAGTTTTATTAG

B

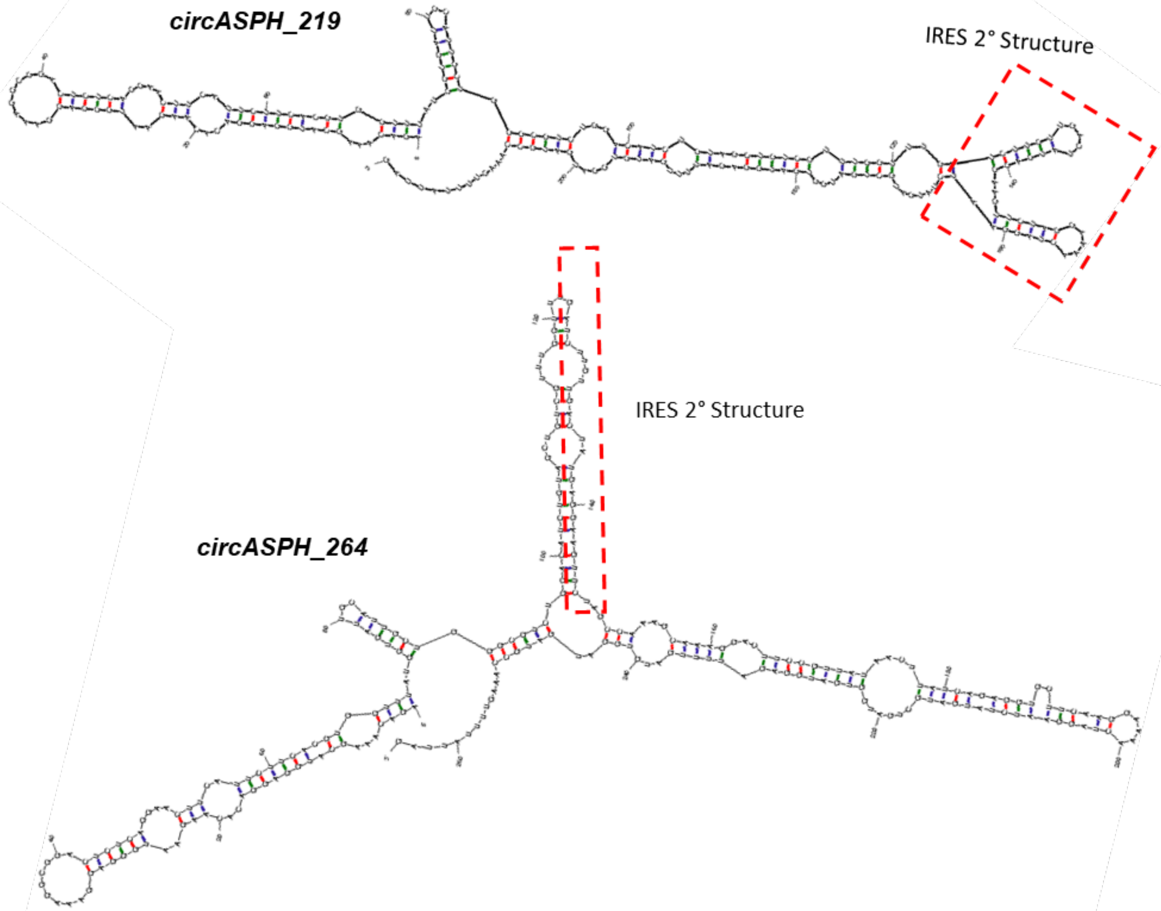

Supplementary Figure S2: **A.** Diagrammatic representation showing biogenesis of *circASPH* splice variants, potential ORFs, and full-length sequences. **B.** Structure predictions of *circASPH_219* and *circASPH_264* using mFold algorithm showing secondary structures of circRNADb database predicted IRES sequence on it.

Supplementary Figure S3

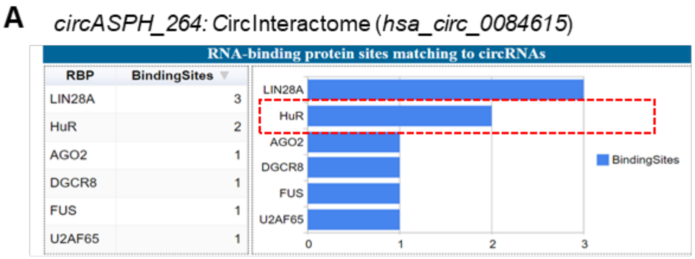

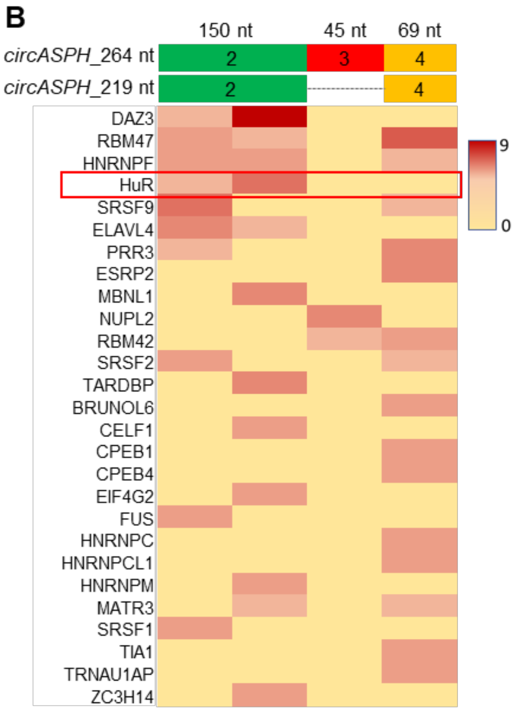

C

circASPH_219		
m6A site Position	Score (combined)	Decision
17	0.576	Low confidence
41	0.643	High confidence
51	0.559	Low confidence

circASPH_264		
m6A site Position	Score (combined)	Decision
17	0.576	Low confidence
41	0.643	High confidence
51	0.559	Low confidence
163	0.597	Moderate confidence

Supplementary Figure S3: A. HuR binding sites on *circASPH_264* (*hsa_circ_0084615*) as reported in circInteractome database. **B.** HuR binding sites on *circASPH* splice variants predicted by RBPmap. **C.** Prediction of m6A sites on *circASPH* splice variants with SRAMP web server.

Supplementary Figure S4

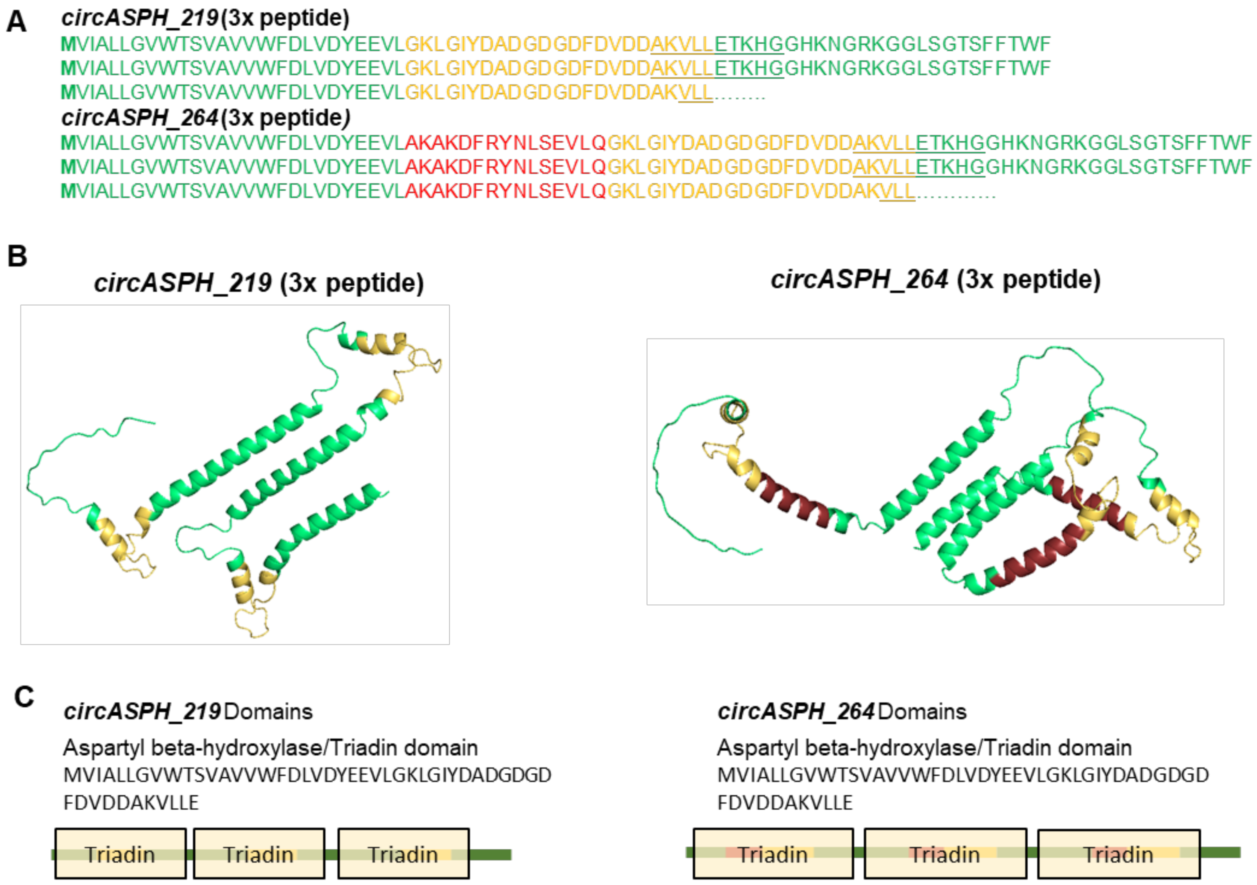

Supplementary Figure S4: Characterization of potential circRNA peptides generated from *circASPH* splice variants. **A.** Peptide sequence generated from different splice variants of *circASPH* with junction peptide sequence. Different colors indicate different exons of the circRNA splice variants, and the underlined sequence marks the backsplice junction sequence. **B.** AlphaFold2 generated potential circRNA peptide structure coming from *circASPH* splice variants. **C.** InterProScan generated domain analysis of the polypeptides generated from *circASPH* splice variants.
